# Facile Fabrication of Polyaniline/Pbs Nanocomposite for High-Performance Supercapacitor Application

**DOI:** 10.3390/nano12050817

**Published:** 2022-02-28

**Authors:** Ahmed Gamal, Mohamed Shaban, Mohammad BinSabt, Mahmoud Moussa, Ashour M. Ahmed, Mohamed Rabia, Hany Hamdy

**Affiliations:** 1 Nanophotonics and Applications Laboratory, Physics Department, Faculty of Science, Beni-Suef University, Beni-Suef 62514, Egypt; a_gamal21@yahoo.com (A.G.); ashour.elshemey@gmail.com (A.M.A.); MOH.RABIE17@yahoo.com (M.R.); hshamdy@hotmail.com (H.H.); 2 Department of Physics, Faculty of Science, Islamic University of Madinah, P.O. Box 170, Al-Madinah Almonawara 42351, Saudi Arabia; 3 Chemistry Department, Faculty of Science, Kuwait University, P.O. Box 5969, Safat 13060, Kuwait; mohammad.binsabt@ku.edu.kw; 4 Future Industries Institute, University of South Australia, Mawson Lakes, SA 5095, Australia; mnasida2002@gmail.com; 5 Chemistry Department, Faculty of Science, Beni-Suef University, Beni-Suef 62511, Egypt

**Keywords:** PANI/PbS nanocomposite, supercapacitor, electrochemical properties, capacitance, stability

## Abstract

In this work, a polyaniline/lead sulfide (PANI/PbS) nanocomposite was prepared by combining the in situ oxidation polymerization method and the surface adsorption process. This nanocomposite was applied as a supercapacitor electrode. The crystal structure, nanomorphology, and optical analysis of PANI and PANI/PbS were investigated. The electrochemical performance of the designed PANI/PbS electrode-based supercapacitor was tested by using cyclic voltammetry (CV), chronopotentiometry (CP), and AC impedance techniques in HCl and Na_2_SO_4_ electrolytes. The average crystallite size of the PANI/PbS nanocomposite is about 43 nm. PANI/PbS possesses an agglomerated network related to PANI with additional spherical shapes from PbS nanoparticles. After the PANI/PbS nanocomposite formation, there are enhancements in their absorption intensities. At a current density of 0.4 A g^−1^, the specific capacitance of PANI/PbS in Na_2_SO_4_ and HCl was found to be 303 and 625 F g^−1^, respectively. In HCl (625 F g^−1^ and 1500 mF cm^−2^), the gravimetric and areal capacitances of the PANI/PbS electrode are nearly double those of the Na_2_SO_4_ electrolyte. Also, the average specific energy and specific power density values for the PANI/PbS electrode in HCl are 4.168 Wh kg^−1^ and 196.03 W kg^−1^, respectively. After 5000 cycles, the capacitance loses only 4.5% of its initial value. The results refer to the high stability and good performance of the designed PANI/PbS as a supercapacitor electrode.

## 1. Introduction

The growing demand for energy storage devices has sparked research into electrochemical supercapacitors (SCs), which have intriguing properties that are similar to both capacitors and batteries. Electrochemical supercapacitors have higher specific energy and specific power than ordinary capacitors and batteries, allowing for faster charging in advanced technology applications [1]. The charge storage techniques of batteries and supercapacitors are the fundamental differences between them. While batteries use the redox reaction to store charge in the bulk of the active material, supercapacitors use the concept of charge storage on the electrode [2]. Depending on the electrode material used in supercapacitors, the charge storage characteristics vary [3]. It is well acknowledged that various types of supercapacitor devices could be used as a helpful storage system for electric energy produced by renewable and alternative energy sources [3]. Concerning the energy storage mechanism, SCs can be classified as electrical double-layer capacitors (EDLCs) and pseudocapacitors. Traditional electrochemical double-layer supercapacitors (EDLCs) have benefits, such as high-power density (in kW kg^−1^) and capability (60–120 s), excellent reversibility (90–95%), and extremely long cycle life (>10^5^) [4]. EDLCs, on the other hand, have low-energy density of approximately 3–5 Wh kg^−1^ [5]. Different pseudocapacitive electrode materials store charge via redox-based Faradaic processes that have been studied to improve energy density [6]. In theory, pseudocapacitive materials can provide a higher energy density than EDLCs (up to 10 Wh kg^−1^) [7]. According to the charge-storing method, pseudocapacitors have a substantially higher energy density and specific capacitance than EDLCs. Pseudocapacitor materials include conducting polymers, metal oxides, and metal sulfides. Among the conducting polymers, polyaniline (PANI) is an excellent material for supercapacitor electrodes due to its high-energy density and fast charge/discharge kinetics [8]. However, conducting polymers have low stability with continuous cycling [9]. As a result, nanocomposites containing metal oxides, such as NiO, MnO_2_, TiO_2_, SnO_2_, Al_2_O_3,_ and V_2_O_5,_ are preferred in supercapacitor electrodes to improve stability during repeated cycles [10,11,12,13,14]. On the other hand, metal sulfides, such as CoS, NiS, ZnS, SnS, and CuS, can be used in energy storage applications, demanding relatively high-power and high-energy densities because of their good conductivity, reliable service lifetime, high stability, and low cost [15,16,17,18,19].

The bandgap of lead sulfide (PbS) is 0.41 eV, the absorption coefficient is 1–5 × 10^5^ cm^−1^, and the Bohr exciton radius is 18 nm [20,21]. It can be used in many applications, such as solar cells, catalysts, sensors, and photodetectors. Few studies have recently investigated how combining PbS with other active materials increases PbS’s energy storage performance [22,23,24,25,26,27,28,29]. For example, Cheol-Hwan Mun et al. synthesized a NiS-PbS composite that exhibits a higher specific capacity of 125.89 mA h g^−1^ at a current density of 2 A g^−1^ [22]. Nasreen et al. developed a Ce_2_Zr_2_O_7_/PbS nanocomposite with a maximum specific capacitance of 219 F g^−1^ at 1 A g^−1^ [23]. Using a one-step method, Yuming et al. developed a fractal fern-like PbS architecture and obtained a high specific capacitance of 498 F g^−1^ [24]. In addition, I. K. Durga et al. synthesized CuS@PbS composite electrode with a high specific capacity of 1004.42 F g^−1^ at a current density of 2.85 A g^−1^ in the three-electrode system [26]. However, to the best of the authors’ knowledge and efforts, there are few reports of nanocomposite materials containing PANI polymer and PbS for supercapacitor applications; thus, the electrochemical performance of SC materials must be improved by the composite PANI/PbS because of the conductivity, stability, and benefit of PbS [30,31,32,33].

In this study, a simple and cost-effective strategy for designing and fabricating a PANI/PbS nanocomposite at room temperature for SC applications was developed. The electrochemical impedance spectroscopy (EIS), cyclic voltammetry (CV), and galvanostatic charge/discharge (GCD) for nanocomposites were measured in various solutions. After 5000 cycles, the electrode materials had a high specific capacitance of 625 F g^−1^ at 0.4 A g^−1^, a specific power of 196.03 W kg^−1^ at 1.735 Wh kg^−1^ specific energy, and capacity retention of 95.5%. These findings point to the PANI/PbS composite’s potential as a long-term performance electrode material for SC applications.

## 2. Experimental Details

### 2.1. Materials

Aniline (C_6_H_5_NH_2_) and ammonium persulfate ((NH_4_)_2_S_2_O_8_) were purchased from Sigma-Aldrich (Gillingham, UK). Nafion (5 wt% in methanol), and dimethyl sulfoxide (DMSO) were purchased from Sigma Aldrich (Missouri, Louis, MO, USA). Hydrochloric acid (HCl), lead nitrate (Pb(NO_3_)_2_), and sodium sulfate (Na_2_SO_4_) were purchased from El-Naser Company (Nasr City, Egypt).

### 2.2. Preparation of PANI Nanopowder

Polyaniline (PANI) was prepared through the in situ oxidation polymerization method. Aniline (0.1 M) was dissolved in 0.5 M HCl under ultrasonic effect. About 0.15 M (NH_4_)_2_S_2_O_8_ dissolved well in distilled water as an oxidant solution. Then, the oxidant was added suddenly over the aniline solution. Over 1 h, a complete precipitate is formed from PANI. The mixture was filtered, washed well, and dried at 60 °C for 12 h to obtain PANI nanopowder.

### 2.3. Preparation of PANI/PbS Nanocomposite

A PANI/PbS nanocomposite was synthesized through the surface adsorption process. A specific weight of PANI nanopowder was thoroughly immersed in a 0.07 M Pb(NO_3_)_2_ solution. Through this process, the adsorption of Pb^2+^ over the PANI surface occurred. Then, PANI/Pb^2+^ was filtrated and dried well. Finally, these dried nanomaterials were immersed in a (0.01 M) thiourea solution at 60 °C for 15 min. The reaction between the Pb^2+^ and S^2−^ led to the formation of the PANI/PbS nanocomposite. This nanocomposite was filtrated, dried, and used for the preparation of the supercapacitor electrode.

### 2.4. Characterization of the Prepared Nanomaterials

The crystal structure of PANI and PANI/PbS was confirmed by X-ray diffraction (XRD; PANalytical, Warsaw Poland) with CuK α radiation (=1.5406A°) at 45 kV and 40 mA. The XRD patterns were taken in the 2θ range of 10–90°. The pattern was examined by comparing the observed peaks to the JCPDS files’ standard patterns. The function groups were identified using a Fourier transform infrared spectrophotometer (FTIR; Jasco, Kyoto, Japan). In addition, the EDX-SEM (Carl ZEISS Sigma 500 VP, Munich, Germany) coupled with the EDX detector at 15 kV and 1243× magnification were used. The elemental composition of the prepared PANI/PbS sample was analyzed using EDX. Scanning electron investigations were also performed using a scanning electron microscope, SEM (Model: ZEISS SUPRA 55 VP and ZEISS LEO, Gemini Column, Munich, Germany) and a transmission electron microscope, TEM (JEOL JEM-2100 TEM, Tokyo, Japan). The oxidation states of the elements were investigated using X-ray photoelectron spectroscopy (XPS), AXIS-NOVA, Kratos Analytical Ltd., Manchester, UK. Finally, the optical examination was performed at room temperature with a UV/Vis/IR spectrophotometer (Lambda 950, Perkin Elmer, Waltham, MA, USA) in the range of 200–1100 nm.

### 2.5. Fabrication of Supercapacitors

In a tiny agate mortar, 20 mg of active material powder and 50 µL of Nafion were disseminated in 300 µL of ethanol and then milled into a slurry. The mixture was stirred for 12 h to obtain a homogeneous catalyst ink. Two identical slurries of roughly 50 µL (3 mg) were mounted on the Au electrode (1 cm^2^). On the other hand, two pieces of filter paper were soaked in electrolytes overnight, which could be either 0.2 M HCl or 0.2 M Na_2_SO_4_. After that, a sheet of filter paper was used as a separator between the two electrodes.

### 2.6. Electrochemical Testing

An electrochemical workstation (CHI 660E; CH Instruments, Austin, TX, USA) was used to perform all electrochemical measurements in two-electrode systems. The measurements included cyclic voltammetry (CV), galvanostatic charge/discharge (GCD), and electrochemical impedance spectroscopy (EIS). The CV tests were performed at different scan rates, ranging from 5 to 80 mV s^−1^ between 0 and 1 V. The GCD measurements were performed at 0.4–3 A g^−1^ in a voltage window between 0 and 1 V. The EIS spectra were obtained at an open circuit potential with 5 mV AC voltage amplitude and frequency from 10 mHz to 100 kHz. All the tests were carried out at room temperature.

## 3. Results and Discussion

### 3.1. Characterization of the Prepared Nanomaterials

#### 3.1.1. XRD Analysis 

**Figure 1:**
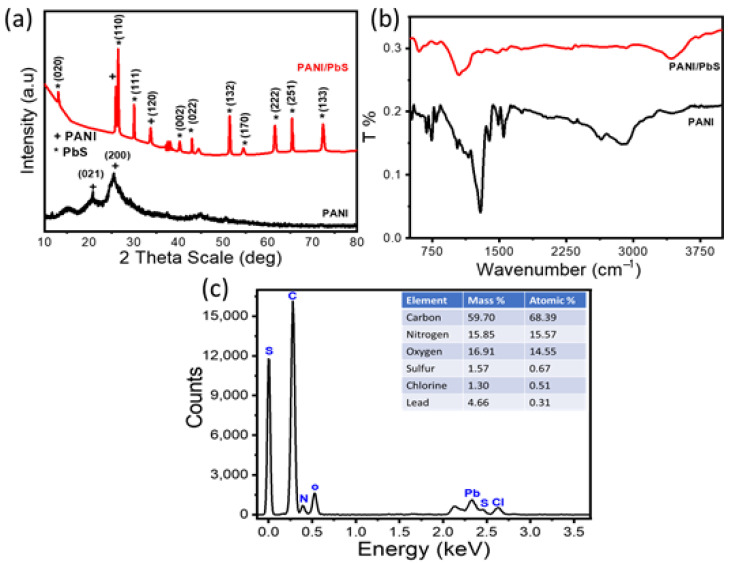
(**a**) XRD, (**b**) FTIR for PANI and PANI/PbS, and (**c**) PANI/PbS EDX pattern with elemental composition percentages.

The crystallographic structures of PANI and PANI/PbS have been confirmed using XRD analyses, as shown in Figure 1a. According to the PANI chart, the semicrystalline nature is confirmed. This is related to the formation of two peaks at 2θ = 20.78°and 25.55° for the growth directions, (021) and (200), respectively, according to JCPDS card no. 53-1891 [34]. The peak at 25.55° is attributed to the parallel periodicity of the polymer chain. There are nine more crystalline peaks related to PbS materials in the PANI/PbS nanocomposite, centered at 2θ = 12.94°, 26.55°, 29.52°, 39.34°, 43.63°, 50.74°, 53.62°, 61.95°, 64.50°, and 72.24°, corresponding to (020), (110), (111), (002), (022), (132), (170), (222), (251), and (133), respectively. This corresponds to the JCPDS card no.05-0592. The peak for PANI at 2θ = 20.78° (021) disappears while 2θ = 25.55° (200) becomes narrow and shaped after the formation of the PANI/PbS nanocomposite. Also, a new peak at 32.32° with a small intensity in PANI/PbS corresponds to the aromatic chain–chain interaction in the PANI. This is due to the interaction and interference between the constituents of the composite [35,36]. This suggests that the nanocomposite formation increases the crystallinity of the PANI. The average crystallite sizes are calculated using Scherrer’s equation based on the full width at half maximum (W in radians), D=0.9 λ/W cosθ [37,38,39,40,41], where λ and θ are the X-ray wavelength (CuK_α_ = 0.15405 nm) and diffraction angle, respectively. By using this equation, the average crystallite size is 24.7 nm for the PANI and increases to 43.4 nm for the PANI/PbS nanocomposite.

#### 3.1.2. FTIR Analysis 

The functional groups of the prepared PANI and PANI/PbS nanocomposite are confirmed by FTIR analyses, as shown in Figure 1b. From this figure, the bands located at 1400 and 1601 cm^−1^ are related to the frequency of PbS heteropolar diatomic molecules, which confirm the formation of PbS [42,43,44]. While the bands at 3401 and 1105 cm^−1^ are related to the formation of the N–H and C–N stretching vibration of NH_2_ of aniline, respectively. The bands at 1467 and 1301 cm^−1^ are related to the C=C vibration of quinoid and benzenoid rings [45,46,47], respectively. After the PANI/PbS composite formation, N–H and C–N bands are red-shifted, whereas the chlorine bands are blue-shifted in comparison with the PANI only. This shift is related to the interaction and formation of the composite constituents [45,46,47,48,49,50]. All the bands of the PANI and PANI/PbS are mentioned clearly in Figure 1b and Table 1 [45,46,47,48,49,50,51,52,53].

#### 3.1.3. EDX Analysis 

The elemental composition of the prepared PANI/PbS sample was determined using an EDX-SEM (Carl ZEISS Sigma 500 VP) coupled with EDS at 15 kV and 1243× magnification. As shown in Figure 1c, the spectrum shows that the composite is primarily made up of carbon, nitrogen, oxygen, sulfur, chlorine, and lead, with mass percentages of 59.70, 15.8, 16.91, 1.57, 1.30, and 4.66. This pattern confirms the polymer construction due to the repeated benzenoid and quinoid rings in the PANI chains, revealing that the main constituents in PANI film are C and N. The lead and sulfur elements are also confirmed through the presence of their peaks. These EDX analyses confirm the formation of the PANI/PbS nanocomposite [54].

#### 3.1.4. XPS Analysis

**Figure 2:**
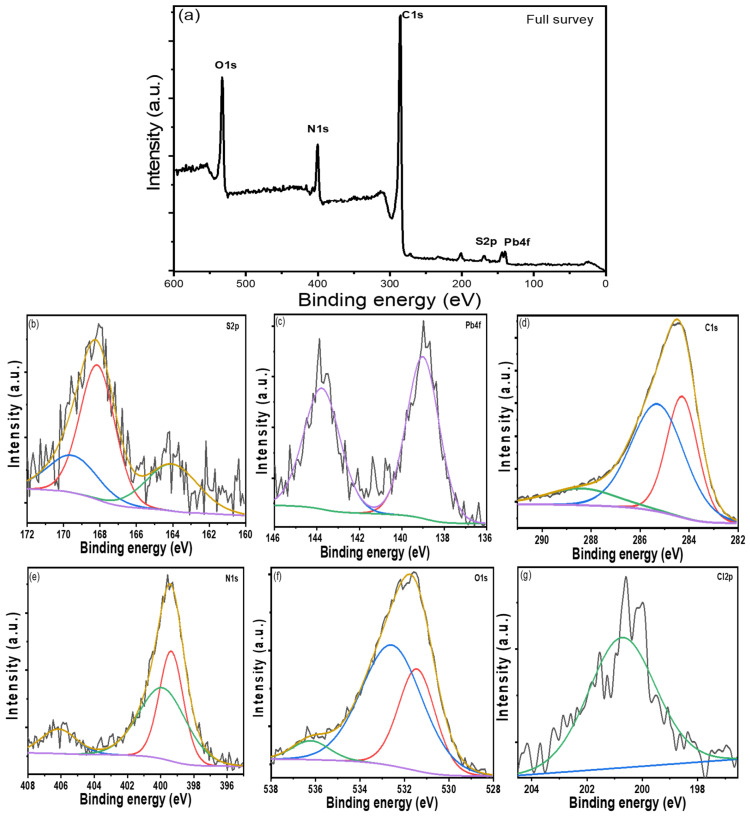
XPS spectra for PANI/PbS sample: (**a**) full survey, (**b**) S 2p region, (**c**) Pb 4f, (**d**) C 1s region, (**e**) N 1s region, (**f**) O 1s region, and (**g**) C1 2p region.

The surface composition and chemical state of the PANI/PbS sample were analyzed by X-ray photoelectron spectroscopy (XPS), AXIS-NOVA, Kratos Analytical Ltd., UK. From XPS survey data, the prepared sample principally consists of oxygen, nitrogen, lead, sulfur, and carbon as presented in Figure 2a. This indicates the formation of a PANI/PbS nanocomposite. The core-level XPS results for the PbS/PANI are shown in Figure 2b–g for the (b) S 2p, (c) Pb 4f, (d) C 1s, (e) N 1s, (f) O 1s, and (g) C1 2p.

Figure 2b shows that the S (2p) region displays many peaks at 164.03, 168.15, and 169.51 eV for the S (2p3/2) spin-orbit-splitting features [55,56]. The S 2p peak at 164.03 eV corresponds to the binding energy of Pb-S [57]. From the Pb 4f core-level spectrum (Figure 2c), the lead contribution is centered at 139.019 eV for the Pb (4f7/2) peak and 143.77 eV for the Pb (4f5/2) peak, corresponding to the Pb^2+^ cations associated with PbS formation [58]. Because the atoms are bound to the more electronegative sulphur of polymer molecules, the core-level-binding energies are higher than those previously reported [59].

In Figure 2d, the C1s XPS spectrum shows a peak at 284.28 eV due to the C-C in the PANI structure [60]. Also, there are two peaks at 285.28 and 288.41 eV attributed to the sp-hybridized carbon [61]. The values of the binding energy (BE) were calibrated based on the C 1s peak (285 eV) as the internal reference line to accurately determine the positions of other spectral lines.

The values of the binding energy (BE) of N1s located at 399.36 and 399.88 eV correspond to cationic species (N^+^) and amine (-NH-) groups for PANI [62]. The N1s spectrum displays the successful polymerization of aniline, as seen in Figure 2e [60]. The core-level oxygen O 1s features in PbS, Figure 2f, are detected at binding energies of 531.44, 532.57, and 536.21 eV, attributed to PbCO_3_ and Pb(OH)_2_ [63]. The adsorption of CO_2_ from the atmosphere onto the PbS surface or the influence of thiourea and polymer from the solution causes the formation of the lead carbonate. The XPS spectrum, Figure 2g, shows one peak at 200.73 eV, which is caused by the binding energy for Cl 2p during aniline polymerization in an acidic medium (HCl).

#### 3.1.5. Morphological Analysis

**Figure 3:**
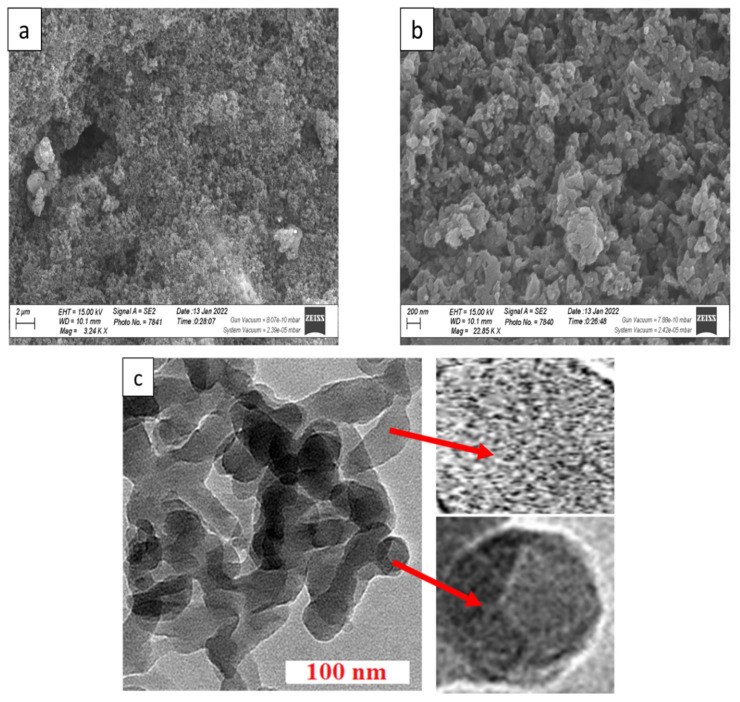
The SEM image of (**a**,**b**) PANI/PbS. (**c**) HR-TEM of PANI/PbS core-shell nano/microcomposite.

The surface morphologies of PANI and PANI/PbS are examined by using SEM images as shown in Figure 3a,b. From Figure 3b, the PANI has randomly distributed nanoparticles that are separated by different voids. This structure has the appearance of a porous network. The sizes of the nanoparticles are ranged from 15 to 150 nm. For the PANI/PbS composite, a similar network shape is observed clearly with additional spherical shapes related to the PbS nanoparticles (Figure 3b). With an average size of 20 nm, PbS nanoparticles have a high density and compact size. This leads to an increase in the surface area of PANI/PbS, which is very useful in storage energy and supercapacitor applications. Figure 3c shows TEM images of the PANI/PbS nanocomposite. The PbS nanoparticles (black color) implanted in PANI (grey color) suggest the creation of a PANI/PbS core-shell nano/microcomposite, as seen in the figure. In the magnified regions of Figure 3c, the spherical and ribbed forms of PbS nanoparticles are visible (bottom inset). The nanoporous nature of the PANI shell is also visible in the magnified regions of Figure 3c (top inset).

#### 3.1.6. Optical Properties

**Figure 4:**
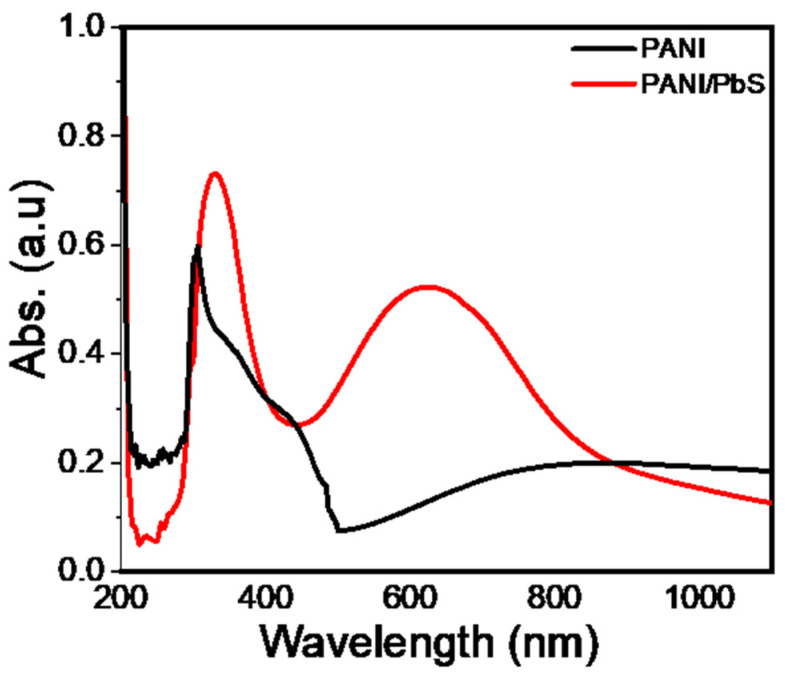
The optical absorbance of PANI and PANI/PbS nanomaterials.

The optical spectra for PANI and PANI/PbS are shown in Figure 4. From this figure, the PANI has an absorption strong peak in the UV region at 302 nm. This peak is related to the benzenoid ring Π-Π* transition. This indicates the successful formation of PANI [64,65]. After the PANI/PbS nanocomposite formation, the two peaks at 332 and 631 nm appeared. Also, there is an enhancement in light-absorption capability. The redshift in peak position from 302 to 332 is due to the interactions of aromatic PANI macromolecules with PbS. The new peaks at 631 nm appear due to the formation of doping levels between PANI and PbS, resulting in various exciton transitions [66].

### 3.2. PANI/PbS Composite Electrochemical Performance

**Figure 5:**
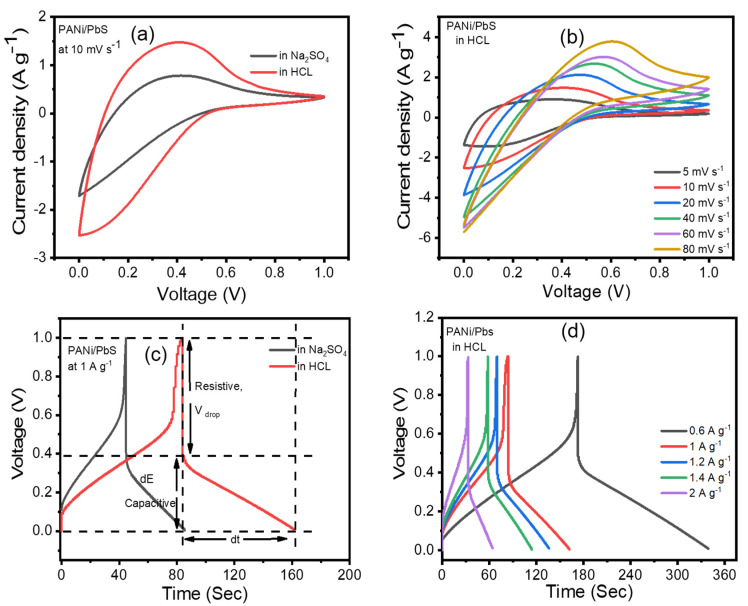
Electrochemical performance of the PANI/PbS electrode: (**a**) CVs in HCl and Na_2_SO_4_ at 10 mV s^−1^ scan rate, (**b**) CVs at different scan rates, (**c**) PANI/PbS charge/discharge curves (CDs) in Na_2_SO_4_ and HCl electrolytes, and (**d**) charge/discharge curves at different current densities.

Cyclic voltammetry (CV), galvanostatic charge/discharge (GCD), and electrochemical impedance spectroscopy (EIS) techniques were used to investigate the electrochemical properties of the PANI/PbS electrode. CV is an electrochemical technique that can reveal a lot about the characteristics of different electrodes in different media [67,68,69]. Figure 5a shows the CV curves of the PANI/PbS in 0.2 M HCl and Na_2_SO_4_ electrolytes at a scan rate of 10 mV s^−1^. It is noted that the area enclosed by the CV curve for HCl electrolyte is significantly larger than that for Na_2_SO_4_. The gravimetric capacitance (C_wt_, F g^−1^) of the fabricated supercapacitor can be calculated from its CV using the formula [70,71,72]:

(1) $$C_{wt}=\frac{4\;\int_{v_{1}}^{v_{n}}i\;dv}{ms\;∆V}\;$$

where $\int_{v_{1}}^{v_{n}}i\;dv$ is the CV curve’s integral area, *s* is the scan rate (V s^−1^), ∆V is (2 × the voltage range, from E_1_ to E_2_ and then back to E_1_) (V), and *m* is the mass of the active materials for both electrodes (g). The calculated gravimetric capacitances (*C_wt_*) of PANI/PbS in Na_2_SO_4_ and PANI/PbS in HCl were found to be 81.5 and 164 F g^−1^, respectively. There are two types of contributions in the composite structure that lead to improvements in electrode capacitive behavior. The structures of the film electrodes are responsible for the electric double-layer capacitance (EDLC) created by PbS, as well as the pseudocapacitive behavior of PANI. Another property of composite electrodes is their ability to maintain current stability. The current in the anodic sweep for PANI/PbS in the HCl electrode reached a maximum at 0.4 V and gradually declined, as shown in Figure 5a. However, with the same style and values, this phenomenon does not occur for PANI/PbS in Na_2_SO_4_.

**Figure 6:**
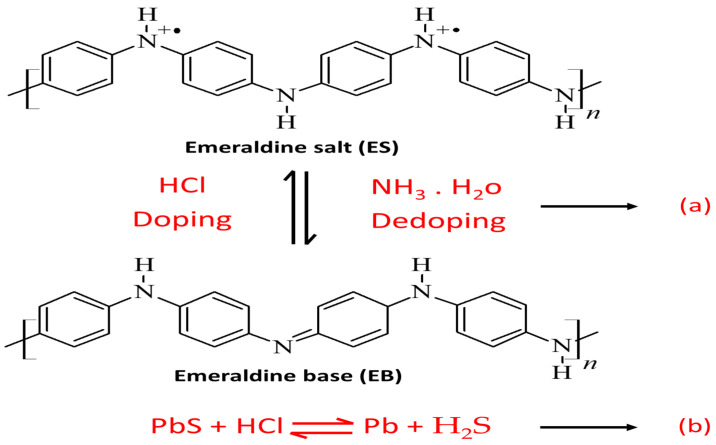
The electrochemical reaction mechanism for (**a**) PANI and (**b**) PbS.

The behavior of supercapacitor electrodes at different scan rates is one of the electrochemical features that demonstrate their kinetic performance. Figure 5b depicts the CV curves of the PANI/PbS electrode in 0.2 M HCl electrolyte at various scan rates, revealing the excellent capacitive performance of the PANI/PbS electrodes according to the electrochemical mechanism depicted in Figure 6a,b. The CV curves of PANI/PbS maintained their shapes by increasing the scan rate until the scan rate reached 2000 mV s^−1^ with little deformation. This can be explained by the PANI/PbS electrode’s perfect capacitive performance [73]. This distortion is caused by the electrolyte and film resistance, and it is dependent on the scan rate. Deeper active sites in composite materials will not have enough time to react with electrolyte ions if the sweep rate is increased.

A galvanostatic charge/discharge (GCD) technique was employed to emphasize the capacitance characteristic of the PANI/PbS electrode. The CD behavior of PANI/PbS in the potential range of 0 to 1 V at a current density of 1 A g^−1^ is shown in Figure 5c. The shape form of this potential range indicates that PANI/PbS as an electrode for supercapacitors has good coulombic efficiency and optimal capacitive behavior. Furthermore, the pseudocapacitance from PANI is to blame for the divergence from linearity. The inclusion of EDLC and faradic capacitance from PbS and PANI, respectively, are responsible for the lengthy charge and discharge times.

The CD curve is made up of two parts: (i) a capacitive component that represents the voltage change caused by the supercapacitor’s energy change; and (ii) a resistive part that represents the voltage change caused by the supercapacitor’s equivalent series resistance (ESR) (as illustrated in Figure 5c). dE is the voltage change during the discharge process, and dt is the discharge period. V_drop_ indicates the voltage drop or voltage fluctuation at the early stage of the discharging curve during the ESR. In general, the appearance of IR drops is ascribed to changes in electrolyte potential, contact resistance, and charge–discharge current density. The lower the conductivity of the electrolyte, the higher the IR drop. The passage of charge carriers (ions) in the electrolyte past the separator to the electrodes and into their porous structure is associated with the charging and discharging of the supercapacitor. The value of IR drop depends also on the current and potential distribution in the electrolyte, which is related to the change in the size or shape of the working electrode. As a result, the IR drop in the charge/discharge curves is considerably larger than the ESR [74,75,76]. The IR drop can be reduced by utilizing a three-electrode system instead of a two-electrode system, providing a high concentration of totally dissociated electrolytes to the solution, scanning at low rates to reduce current, reducing electrode surface area, and positioning the reference electrode tip near the working electrode surface.

The CD curves of the PANI/PbS electrode at different current densities are shown in Figure 5d. As shown, increasing the current density reduced the magnitude of the specific capacitance (SC) of the PANI/PbS electrode because there is not sufficient time for ion intercalation at the surface of the active material in the electrode/electrolyte interface.

**Figure 7:**
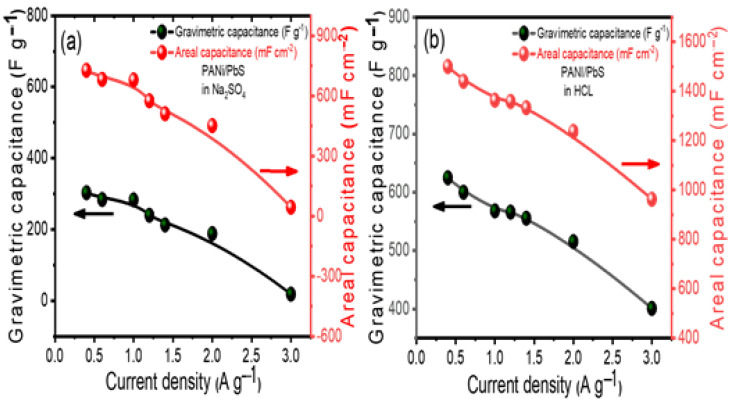
Electrochemical performance of PANI/PbS: (**a**) calculated areal and gravimetric capacitances for PANI/PbS in Na_2_SO_4_ at various current densities, and (**b**) calculated areal and gravimetric capacitances for PANI/PbS in HCl electrolyte at various current densities.

The gravimetric (*C_wt_*) and areal (*C_A_*) capacitances of the supercapacitor at various current densities are shown in Figure 7a,b, as derived from CD curves using the following formulae [70,71,72]:

(2) $$C_{wt}\;=\;\frac{4I}{m\left(\frac{∆E}{∆t}\right)}$$

(3) $$C_{A}\;=\;\frac{4I}{A\left(\frac{∆E}{∆t}\right)}$$

where *I* is the constant current applied (A) and $\left(\frac{∆E}{∆t}\right)$ is the discharge curve’s slope. The total mass of the two electrodes is given by *m* (g), and the footprint area (cm^2^) of the two electrodes is denoted by *A*.

Gravimetric capacitances, as opposed to volumetric and areal values, have been proven to give a more realistic depiction of a supercapacitor’s genuine performance. This is especially true in the case of this electrode, as the active material mass is much larger than in micro-devices. As a result, we estimated the specific capacitance of this electrode based on the active material’s weight. Figure 7a,b depicts the material’s capacitance as a function of varying current densities. The gravimetric and areal capacitances (*C_wt_* and *C_A_*) of the PANI/PbS electrodes in the Na_2_SO_4_ and HCl electrolytes at various current densities are shown in Figure 7a,b. The highest values of the *C_wt_* are 625 F g^−1^ for the HCl electrolyte and 303.03 F g^−1^ for the Na_2_SO_4_ electrolyte at a current density of 0.4 A g^−1^. Similarly, the areal capacitance per footprint rises from 962.57 mF cm^−2^ to 1500 mF cm^−2^ for the HCl electrolyte and from 44.1 mF cm^−2^ to 727.27 mF cm^−2^ for the Na_2_SO_4_ electrolyte as the current density decreases. The high gravimetric and areal capacitances performance in HCl compared to Na_2_SO_4_ can be ascribed to the following two reasons: (1) the migration speed of H^+^ ions in the bulk electrolyte and the inner pores of the electrode is greater than the migration speed of Na^+^ and the speed of Cl^−^ is greater than the speed of SO_4_^2−^, and (2) the equivalent series resistance in the HCl is less than the equivalent series resistance in the Na_2_SO_4,_ as discussed in the following paragraph, which is the main factor influencing the maximum output power.

**Figure 8:**
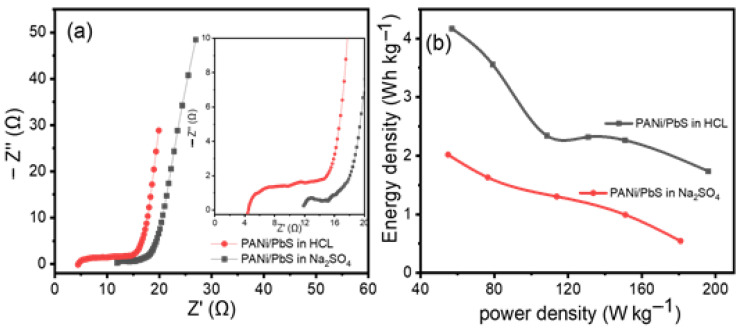
(**a**) PANI/PbS Nyquist plots in Na_2_SO_4_ and HCl. The amplified Nyquist plots in the high-frequency area are shown in the inset. (**b**) The Ragone plot shows the specific power vs. specific energy for PANI/PbS in Na_2_SO_4_ and HCl, respectively.

To fully comprehend our material’s electrochemical behavior, electrochemical impedance spectroscopy (EIS) is required. The Nyquist plots of the material electrodes tested in HCl and Na_2_SO_4_ electrolytes are shown in Figure 8a. In the high-frequency range, the sample in HCl has a more pronounced semicircle (inset in Figure 8a). We calculated an equivalent series resistance (ESR) of 11.89 Ω for the electrodes in Na_2_SO_4_ by extrapolating the straight region of the graph, which is higher than that obtained in HCl electrolyte (4.414 Ω). At low frequencies, the material in HCl has a more vertical line than the material in Na_2_SO_4_, indicating that the HCl electrolyte has a lower diffusion resistance of ions and a higher capacitance.

Using the following equations, we created the Ragone plot in Figure 8b with the specific energy (*E_wt_*) and specific power (*P_wt_*) based on the total mass of electroactive materials in the two electrodes [70,71,72].

(4) $$E_{wt}\left(\mathrm{Wh}\;g^{-1}\right)\;=\;A\;C_{wt}{\left(∆E\right)}^{2}$$

(5) $$P_{wt}\left(W\;g^{-1}\right)\;=\;B\frac{E_{wt}}{∆t}$$

where *A* is a constant equal to $\frac{0.125}{3.6}$, and *B* is a constant equal to 3600.

The average specific energy and specific power values for the PANI/PbS electrodes in Na_2_SO_4_ are 2.02 Wh kg^−1^ and 180.89 W kg^−1^, respectively. Much higher values of 4.168 Wh kg^−1^ and 196.03 W kg^−1^ are found for the HCl electrolyte. Such a configuration could be characterized as a supercapattery cell based on the specific energy and power obtained [77]. Ragone plots are provided as supplementary data (Figure S1) to compare the specific energy for our electrode with the previously reported data in the literature to show the merits of our PANI/PbS electrode relative to the previously reported data.

**Figure 9:**
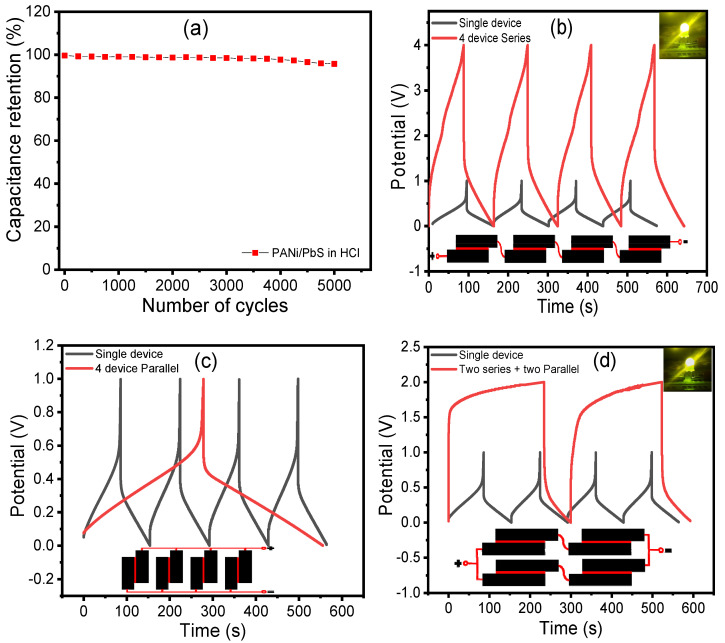
(**a**) The electrode’s performance stability. The electrode retains 95.5% of its initial capacitance after 5000 cycles. Charge/discharge curves for four tandem micro-supercapacitors connected (**b**) in series, (**c**) in parallel, and (**d**) in a series–parallel configuration. For comparison, only one gadget is shown. Both the tandem and single configurations were charged and discharged at the same current. (Insets) A light-emitting diode can be powered by a tandem supercapacitor (LED).

The cycling performance of the cell was investigated for 5000 cycles (Figure 9a), with a current of 3 mA (1 A g^−1^) in HCl electrolyte and a charge/discharge cycle period of 163.8 s. The obtained specific capacitance’s dependence on cycle number are plotted. As a result, it is possible to estimate that after approximately 5000 cycles, a capacitance loss of 4.5% will be obtained from an initial value of 568.2 F g^−^^1^ at 1 A g^−1^. The estimated cycle counts for pure polyaniline electrodes [78,79,80,81] and pure lead sulfide electrodes [64] were in good agreement.

As a result of ion doping–dedoping, pure polyaniline electrodes exhibit significant volumetric swelling and shrinking throughout the charge/discharge process [82]. This volumetric exchange frequently results in structural failure and, as a result, relatively rapid capacitance degradation. After over 1000 cycles, most polyaniline and polypyrrole-based electrodes appear to retain less than half of their initial capacitance. As a result, cycle instability is a significant barrier to pure conductive polymer electrodes being used in practical applications. Liu et al. [83] recently improved the stability of polyaniline and polypyrrole electrodes by building a thin carbonaceous shell onto the polymer surface via a hydrothermal reaction utilizing glucose as a carbon precursor. Carbonaceous shell-coated polyaniline and polypyrrole electrodes retained 95% and 85% of their capacitance after 10,000 cycles, respectively. On the other hand, after approximately 450–500 cycles of pure lead sulfide at deep discharge, 100 DOD, 80% of the capacity was lost [84]. By incorporating a small amount of different carbon materials into the active mass of the lead sulfide electrode, this might be greatly improved [85,86]. The addition of carbon compounds to the cell could potentially improve the cell’s resistance.

In general, the total energy that a single supercapacitor can store is insufficient for most practical uses. As a result, supercapacitors must be connected in series and/or parallel, just like batteries, to form a ‘bank’ with a certain voltage and capacitance rating, depending on the application. Connecting four configurations in series and parallel configurations, as shown in Figure 9b–d, demonstrates the versatility of the PANI/PbS electrode for serial and parallel combinations. The tandem PANI/PbS electrode has excellent control over the operating voltage window and capacity, making it suitable for practical applications. The tandem devices, like the individual supercapacitors, have practically perfect triangular charge/discharge curves with a small voltage drop, indicating good capacitive characteristics with little internal resistance. This outstanding performance was obtained without the use of voltage balancing, which is commonly used with series connections to prevent any cell from going into over-voltage.

## 4. Conclusions

Finally, by combining the in situ oxidation polymerization approach with the surface adsorption process, a PANI/PbS nanocomposite was created. This composite was used in the fabrication of the supercapacitor electrode, PANI and PANI/PbS crystal structure, nanomorphology, and optical analysis. Utilizing HCl and Na_2_SO_4_ electrolytes, the electrochemical performance of the developed PANI/PbS electrode-based supercapacitor was investigated using cyclic voltammetry, chronopotentiometry, and electrochemical AC impedance techniques. The PANI/PbS nanocomposite has an average crystallite size of 43 nm. PANI/PbS has an agglomerated nanoparticulate porous network similar to PANI, in addition to spherical PbS nanoparticles. There are redshifts in the peaks of 330 and 630 nm after the PANI/PbS composite production, as well as increases in their absorption strengths. The specific capacitances of PANI/PbS in Na_2_SO_4_ and HCl were determined to be 303 and 625 F g^−1^, respectively. Due to the incorporation of electrochemical double-layer capacitance and faradic capacitance from PbS and PANI, respectively, the application of PANI/PbS as an electrode for a supercapacitor has good coulombic efficiency, ideal capacitive behavior, and lengthy charge and discharge times. The gravimetric and areal capacitances of the PANI/PbS electrode in HCl (625 F g^−1^ and 1500 mF cm^−2^) are nearly twice as large as those in the Na_2_SO_4_ electrolyte. For the PANI/PbS electrode in HCl, the average specific energy and specific power values are 4.168 Wh kg^−1^ and 196.03 W kg^−1^, respectively. The capacitance loses 4.5% of its initial value after about 5000 cycles, indicating the remarkable stability of the specified PANI/PbS electrode.

## Figures and Tables

**Table 1:** The FTIR analyses for PANI and PANI/PbS nanomaterials.

Band Position (cm^−1^)		Assignment
PANI	PANI/PbS	
3401	3424	N–H stretching vibrations of amino groups in PAN
2918	2924	The vibration of the C–H aromatic ring
1561	2858	The coordinated water molecule
1467	1590	C=C stretching vibrations of the quinoid ring
-	1460	Frequency of heteropolar diatomic molecules of PbS
1301	1384	C=C vibration of benzenoid rings
1105	1289	C–N stretching vibrations
1049	1121	Frequency of heteropolar diatomic molecules of PbS and chloride group incorporation in the polymer chain
789	1049	C–H in-plane bending vibration
587	793	Para disubstituted aromatic rings

## Data Availability

The data presented in this study are available on request from the corresponding author.

## Supplementary Materials

The following supporting information can be downloaded at https://www.mdpi.com/article/10.3390/nano12050817/s1, Figure S1: The Ragone plot shows the specific energy vs. specific power for the PANI/PbS in HCl and the PANI/PbS with Na_2_SO_4_ [5,23,24,26,28,87].
