# Supplementary material for: Facile Fabrication of Polyaniline/Pbs Nanocomposite for High-Performance Supercapacitor Application
Source: Nanomaterials (Basel). 2022 Feb 28;12(5):817. doi: 10.3390/nano12050817 (PMC8912390; doi:10.3390/nano12050817)
Supplement: Supplementary file 1 [file nanomaterials-12-00817-s001.zip › nanomaterials-1548440-supplementary.pdf]

Supplementary Materials

Facile Fabrication of Polyaniline/Pbs Nanocomposite for High-Performance Supercapacitor Application

Ahmed Gamal [1], Mohamed Shaban [1,2,*], Mohammad BinSabt [3], Mahmoud Moussa [4,5], Ashour M. Ahmed [1], Mohamed Rabia [1,5] and Hany Hamdy [1]

[1] Nanophotonics and Applications Laboratory, Physics Department, Faculty of Science, Beni-Suef University, Beni-Suef 62514, Egypt; a_gamal21@yahoo.com (A.G.); ashour.elshemey@gmail.com (A.M.A.);
MOH.RABIE17@yahoo.com (M.R.); hshamdy@hotmail.com (H.H.)

[2] Department of Physics, Faculty of Science, Islamic University of Madinah, P.O. Box 170, Al-Madinah Almonawara 42351, Saudi Arabia

[3] Chemistry Department, Faculty of Science, Kuwait University, P.O. Box 5969, Safat 13060, Kuwait; Mohammad.binsabt@ku.edu.kw

[4] Future Industries Institute, University of South Australia, Mawson Lakes, SA 5095, Australia; mnasida2002@gmail.com

[5] Chemistry Department, Faculty of Science, Beni-Suef University, Beni-Suef 62511, Egypt

* Correspondence: mssfadel@aucegypt.edu

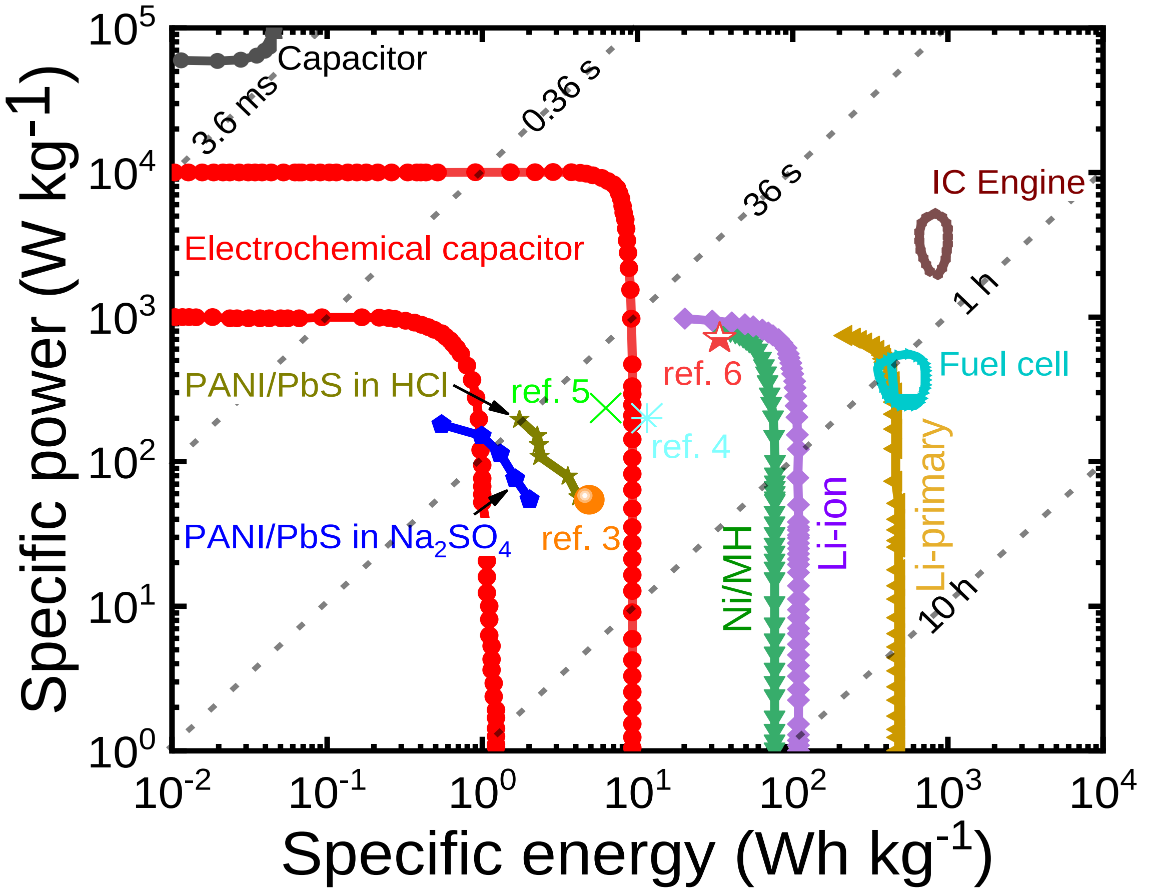

Figure S1. The Ragone plot shows the specific energy vs. specific power for the PANI/PbS in HCl and the PANI/PbS with Na_2SO_4

References

1. Simon, Patrice, and Yury Gogotsi. "Materials for electrochemical capacitors." *Nanoscience and technology: a collection of reviews from Nature journals*. **2010**. 320-329.
2. McCloskey, Bryan D. "Expanding the Ragone plot: Pushing the limits of energy storage." *The journal of physical chemistry letters* 6.18 (**2015**): 3592-3593.
3. Chaudhary, Nahid, and Manika Khanuja. "High-Performance Supercapacitor Electrode Material Based on the Two-Dimensional/Three-Dimensional Architecture of MoS2–PbS Hybrid Material." *Energy & Fuels* (2021).
4. Dai, Yuming, et al. "Fractal fern-like PbS hierarchical architectures for supercapacitors with excellent long-term cycling stability." *Journal of Alloys and Compounds* 805 (**2019**): 631-637.
5. Bibi, Nasreen, et al. "Mesoporous Ce2Zr2O7/PbS nanocomposite with an excellent supercapacitor electrode performance and cyclic stability." *ChemistrySelect* 4.2 (**2019**): 655-661.
6. Kanaka Durga, I.; Srinivasa Rao, S.; Ahn, J.-W.; Park, T.-Y.; Jin-Soo, B.; Ho, C.-I.; Prabakar, K.; Kim, H.-J. Dice-like nanostructure of a CuS@ PbS composite for high-performance supercapacitor electrode applications. *Energies* **2018**, *11*, 1624.
